# Olfactory neuroblastoma with orbital protrusion arising in the background of maxillary carcinoma

**DOI:** 10.1093/jscr/rjae484

**Published:** 2024-08-21

**Authors:** Nikhar Wadhwani, Nitin Bhola

**Affiliations:** Department of Oral and Maxillofacial Surgery, Sharad Pawar Dental College, Datta Meghe Institute of Higher Education and Research, Sawangi, Wardha, Maharashtra (442001), India; Department of Oral and Maxillofacial Surgery, Sharad Pawar Dental College, Datta Meghe Institute of Higher Education and Research, Sawangi, Wardha, Maharashtra (442001), India

**Keywords:** olfactory neuroblastoma, esthesioneuroblastoma, nasal cavity, malignant tumor, metastasis

## Abstract

An uncommon entity in the class of malignant neuroectodermal nasal tumors is the olfactory neuroblastoma, which originates in the roof of the nasal cavity from the olfactory epithelium. It is often mistaken by clinicians for a nasal polyp because it presents with indistinct features such as nasal obstruction and secondary sinus disease. Olfactory neuroblastoma has been observed to cause morbidity by distant metastasis, invasion through the cribriform plate, and secondary meningitis in most instances. It exhibits a range of biologic activities, from slow growth accompanied by long-term patient survival to a very aggressive malignancy with extensive metastases. We report the incidence of a rare case in which a patient, previously operated on and irradiated for squamous cell carcinoma of the maxilla, developed an olfactory neuroblastoma with orbital protrusion.

## Introduction

Olfactory neuroblastoma is a rare malignant entity affecting the olfactory neuroepithelium, and can spread to the base of the skull or remain limited to the cribriform plate [1]. The tumor is generally regarded to be slow-growing, although its aggressiveness varies. Olfactory neuroblastoma displays a bimodal distribution, showing no gender bias, and typically manifests between the second and sixth decades of life [2]. They are presumed to originate from cells of the neural crest and exhibit a wide array of clinical symptoms, along with pathologically mimicking a variety of tumors [3]. This case report aims to provide insights into the clinical course, diagnosis, and management of a patient with olfactory neuroblastoma.

## Case presentation

A 55-year-old male reported with the complaint of blood-stained discharge from the right nostril for 6 months, associated with a slow-growing swelling over the right infraorbital region. There was no accompanying paresthesia, anosmia, or any visual disturbances. He reported no prior experiences of headaches, dizziness, or diplopia. The patient had history of squamous cell carcinoma of the right maxillary region, for which he underwent a right-sided maxillectomy in 2019, followed by 30 fractions of radiotherapy. The patient had a post-surgical defect in the maxilla that resulted in depression near the right maxillary region.

On examination, a swelling approximately 2 × 1.5 cm^2^ in largest dimension, was present over right lateral infraorbital region, firm in consistency and fixed to underlying tissues. Direct nasal endoscopy revealed an unobstructed nasal cavity bilaterally with no visible mass, but excessive secretions in the right side. There were no enlarged lymph nodes detected in the neck.

**Figure 1:**
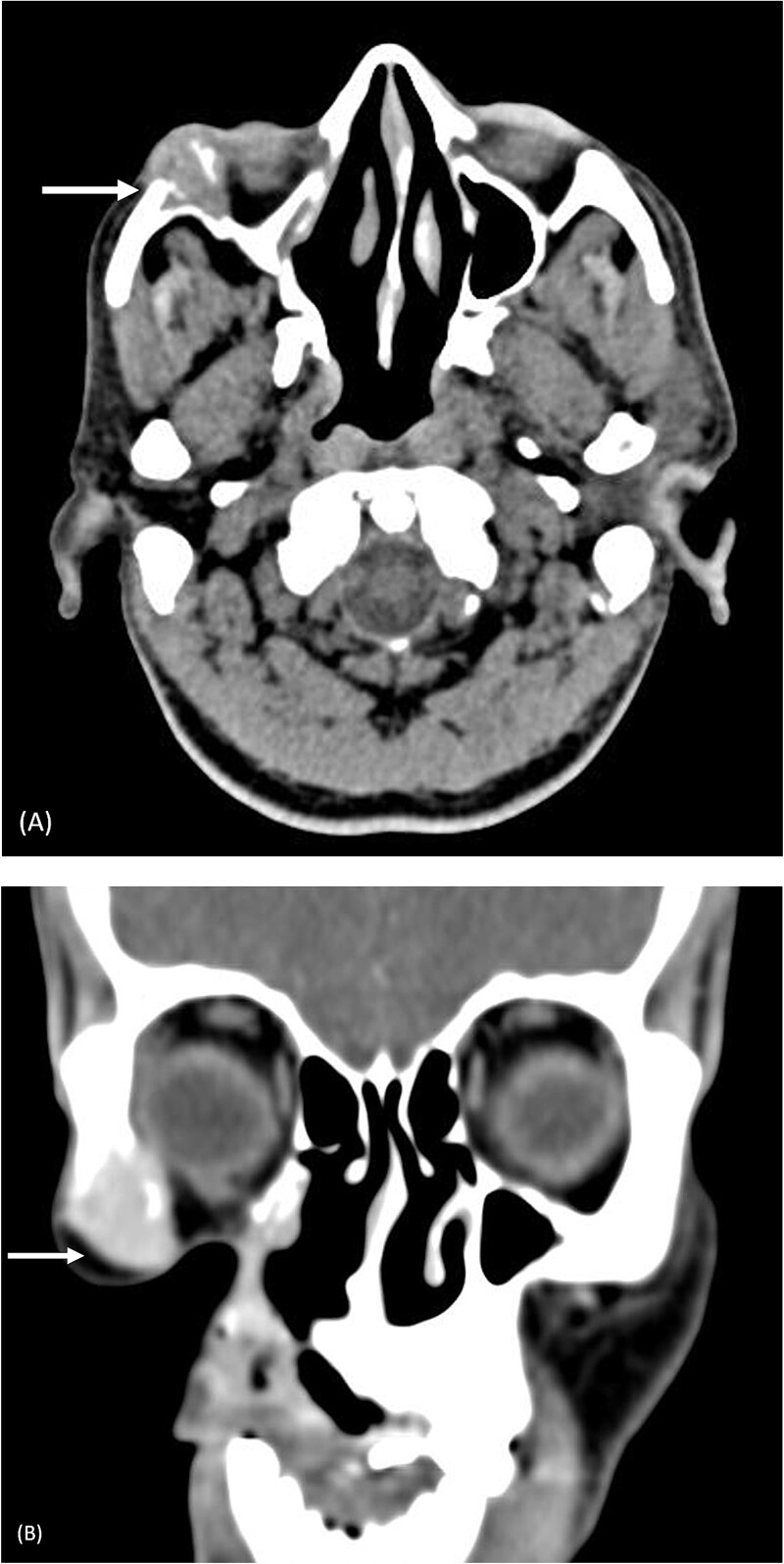
Pre-operative computed tomography of the zygomatic region. (A) Axial CT (bone algorithm) demonstrating a mass of size 2.2 × 2.1 × 2.1 cm^3^ in the right infraorbital region; (B) coronal CT (bone algorithm) demonstrating erosion of the lateral wall of the right orbit and right zygomatic bone, with involvement of the lateral rectus muscle.

Fine needle aspiration cytology of the swelling revealed a malignant round cell tumor that was given the differential diagnosis of olfactory neuroblastoma. On computed tomography (CT), the lesion was causing erosion of the lateral wall of the right orbit and right zygomatic bone, with involvement of the lateral rectus muscle, shown in Fig. 1.

**Figure 2:**
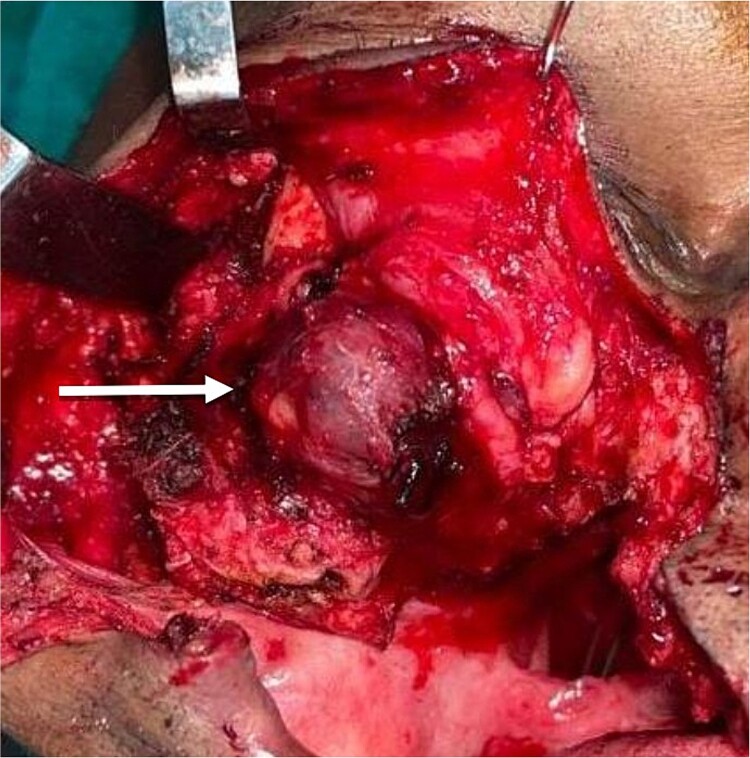
Visible tumor mass after reflection of flap.

The planned treatment was local excision under general anesthesia. For resection of the tumor, a Weber–Fergusson incision was taken from the pre-existing surgical scar over the right side. The tumor mass was resected along with the lateral wall of the orbit of the right side, the floor of the orbit, and the right zygomatic arch, shown in Fig. 2. Standard marking for the hemicoronal incision over the right side was done for harvesting the temporalis myofascial flap. The flap was tunneled under the floor of the orbit and sutured over soft tissue on the medial wall of the orbit. Closure was done after securing a minivac drain.

Histopathological examination of the tumor specimen concluded the diagnosis of olfactory neuroblastoma.

For post-operative management, a supine position was advised with 20° head elevation. Manoeuvres that may result in straining the nasal cavity, such as nose-blowing or leaning forward, were to be avoided. The patient was advised postoperative radiotherapy after complete healing of the surgical site.

## Discussion

Also referred to as esthesioneuroblastoma, olfactory neuroblastoma is a malignant tumor that emerges from the olfactory epithelium in the nasal vault, originating from neural crest cells. This tumor is challenging to comprehend fully due to its rarity. It possesses the capability to infiltrate adjacent structures, including the anterior skull base, orbit, and ethmoidal sinus [4]. In some cases, it can extend to the opposite nasal cavity by crossing the midline. The clinical presentation of olfactory neuroblastoma varies, commonly featuring unilateral nasal obstruction (observed in 70% of cases) and epistaxis (occurring in 50% of cases) [5]. Additional symptoms encompass anosmia, headache, pain, excessive lacrimation, and rhinorrhea.

A noteworthy rarity involves cases where the tumor is centered in the orbit, leading to prominent orbital protrusion. Olfactory neuroblastoma can exhibit periorbital extension, resulting in proptosis, periorbital edema, and diminished visual acuity [6]. Less commonly, olfactory neuroblastoma may be linked to the syndrome of inappropriate antidiuretic hormone secretion, resulting in dilutional hyponatremia. It might lead to Cushing syndrome through the production of ectopic adrenocorticotropic hormone.

Kadish *et al.* and Dulguerov *et al.* were the pioneers in suggesting a staging classification for this tumor. Morita *et al.* made modifications to the Kadish staging system by introducing four groups. This is used as the predominant method for categorizing the anatomic extent of the tumor [7]. Based on the modified Kadish staging, the case in question falls into group C, showing spread of tumor beyond the nasal cavity and paranasal sinuses, reaching the orbit.

Diagnosing olfactory neuroblastoma presents challenges due to its histological resemblance to various sinonasal tumors, but can be differentiated by the presence of clusters of uniform cells with unclear boundaries and a backdrop of fibrous material, and Homer-Wright pseudorosettes [8]. CT is crucial as an initial study for lesion identification, especially in assessing bony involvement. On the other hand, MRI excels at evaluating the extent of soft tissue invasion. Typically, olfactory neuroblastoma appears as a homogeneous mass with necrotic, nonenhancing areas on CT [9]. Characteristic features include a mass across the cribriform plate, typically in the shape of a dumbbell, and cysts surrounding the mass at the skull-tumor interface. This combination of imaging modalities aids in achieving a more accurate and detailed understanding of the nature and extent of the tumor.

In this particular case, a temporalis flap was used for the reconstruction of the defect post-resection. The temporalis muscle flap’s versatility allows it to encompass a range of tissue types, including skin, bone, fascia, periosteum, and muscle. Renowned for its reliability and predictability, the flap boasts a well-defined vascular pedicle, and the feeding vessels can be easily identified and preserved, contributing to its reputation as a durable option with an exceptionally low failure rate. Furthermore, the temporalis muscle flap’s ample length and flexible arc of rotation make it particularly well suited for addressing defects in various anatomical regions [10].

This case report emphasizes the importance a multidisciplinary approach in treating olfactory neuroblastoma. With timely intervention and comprehensive care, patients with this rare tumor can achieve favorable outcomes and maintain a good quality of life.

## Declaration

The authors declare that the work described has not been published previously and that it is not under consideration for publication elsewhere, that its publication is approved by all authors and tacitly or explicitly by the responsible authorities where the work was carried out, and that, if accepted, it will not be published elsewhere including electronically in the same form, in English or in any other language, without the written consent of the copyright-holder.

## Conflict of interest statement

None declared.

## References

[1] Fiani B , Quadri SA, Cathel A, et al. Esthesioneuroblastoma: a comprehensive review of diagnosis, management, and current treatment options. World Neurosurg 2019;126:194–211. 10.1016/j.wneu.2019.03.014. 30862589

[2] Bradley PJ , Jones NS, Robertson I. Diagnosis and management of esthesioneuroblastoma. Curr Opin Otolaryngol Head Neck Surg 2003;11:112–8. 10.1097/00020840-200304000-00009. 14515089

[3] Romani C , Bignotti E, Mattavelli D, et al. Gene expression profiling of olfactory neuroblastoma helps identify prognostic pathways and define potentially therapeutic targets. Cancer 2021;13:2527. 10.3390/cancers13112527. PMC8196700 34064009

[4] Diaz EM , Johnigan RH, Pero C, et al. Olfactory neuroblastoma: the 22-year experience at one comprehensive cancer center. Head Neck 2005;27:138–49. 10.1002/hed.20127. 15654688

[5] Dulguerov P , Allal AS, Calcaterra TC. Esthesioneuroblastoma: a meta-analysis and review. Lancet Oncol 2001;2:683–90. 10.1016/S1470-2045(01)00558-7. 11902539

[6] Chen L , Wang J, Yang Z, et al. Olfactory neuroblastoma of the sinonasal tract with prominent orbital protrusion: a case report and literature review. Indian J Otolaryngol Head Neck Surg Off Publ Assoc Otolaryngol India 2022;74:1281–6. 10.1007/s12070-020-02359-x. PMC9702098 36452830

[7] Morita A , Ebersold MJ, Olsen KD, et al. Esthesioneuroblastoma: prognosis and management. Neurosurgery 1993;32:706–14 discussion 714-715. 10.1227/00006123-199305000-00002. 8492845

[8] Shah K , Perez-Ordóñez B. Neuroendocrine neoplasms of the sinonasal tract: neuroendocrine carcinomas and olfactory neuroblastoma. Head Neck Pathol 2016;10:85–94. 10.1007/s12105-016-0696-7. 26830400 PMC4746139

[9] Su SY , Bell D, Hanna EY. Esthesioneuroblastoma, neuroendocrine carcinoma, and sinonasal undifferentiated carcinoma: differentiation in diagnosis and treatment. Int Arch Otorhinolaryngol 2014;18:S149–56. 10.1055/s-0034-1390014. 25992139 PMC4399581

[10] Cenzi R , Carinci F. Calvarial bone grafts and temporalis muscle flap for midfacial reconstruction after maxillary tumor resection: a long-term retrospective evaluation of 17 patients. J Craniofac Surg 2006;17:1092–104. 10.1097/01.scs.0000246505.86721.54. 17119411

